# Weight Loss Strategies in Male Competitors of Combat Sport Disciplines

**DOI:** 10.3390/medicina57090897

**Published:** 2021-08-28

**Authors:** Alejandro Martínez-Rodríguez, Néstor Vicente-Salar, Carlos Montero-Carretero, Eduardo Cervelló-Gimeno, Enrique Roche

**Affiliations:** 1 Department of Analytical Chemistry, Nutrition and Food Sciences, Faculty of Sciences, University of Alicante, San Vicente del Raspeig, 03609 Alicante, Spain; amartinezrodriguez@gcloud.ua.es; 2 Alicante Institute for Health and Biomedical Research (ISABIAL), 03010 Alicante, Spain; nvicente@umh.es; 3 Department of Applied Biology-Nutrition, Institute of Bioengineering, University Miguel Hernández, 03202 Elche, Spain; 4 Sport Research Center, Department of Sport Sciences, University Miguel Hernández of Elche, 03202 Elche, Spain; cmontero@umh.es (C.M.-C.); ecervello@umh.es (E.C.-G.); 5 CIBER Fisiopatología de la Obesidad y Nutrición (CIBEROBN), Instituto de Salud Carlos III (ISCIII), 28029 Madrid, Spain

**Keywords:** body weight, eating disorders, judo, karate, taekwondo, weight management

## Abstract

*Background and objective*: The use of suboptimal weight loss strategies in order to reach specific weight ranges as observed in combat sport disciplines can give rise to severe health problems. However, particular aspects regarding management of weight category comparing three sport disciplines remain to be investigated. Therefore, the aim of the present study was to obtain information regarding the weight loss strategies that competitors performed before a tournament. *Materials and Methods*: This article describes the most common dietary-nutritional strategies used by 140 national university male competitors of judo (*n* = 52), karate (*n* = 40) and taekwondo (*n* = 48) in order to achieve a specific weight, according to the rapid weight loss questionnaire (RWLQ) and the EAT-27 questionnaire. *Results:* Around 50% of participants were not involved in a weight loss process. Among the remaining participants, we considered three periods for weight reduction: less than 1 week (35% in judo, 8% in karate and 19% in taekwondo), less than 1 month (17% in judo, 15% in karate and 26% in taekwondo) and more than 1 month (0% in judo, 5% in karate and 21% in taekwondo). Severe fasting, focused on food/water restriction, was the most commonly used strategy, being more frequent in judo players. Light weight judo practitioners generally lost 2–5 kg before the contest. One third of participants avoided carbohydrate consumption when performing food restriction. Finally, individuals that reduced weight in the last week seemed to develop an unhealthy psychological relationship with food. *Conclusion*: All these aspects could be particularly relevant, providing information regarding how competitors manage basic nutritional concepts that guide dieting strategies. This information is relevant to prepare future educational interventions in the area of nutrition for competitors, coaches and technical staff.

## 1. Introduction

The three combat sports that derive from East Asian martial arts (judo, karate and taekwondo) were together for the first time as Olympic disciplines in Tokyo 2020. A common aspect of these sports is that the participants compete based on their weight category. For this reason, weight management is of instrumental importance for an optimal physical and psychological performance in these disciplines [1,2]. Weight categories are used in order to match competitors with similar physical characteristics. In this context, top skills and performance expect to be the differential factors to win the combat. Unfortunately, the negative aspect of this system is the unhealthy methods used by competitors to reach the weight category [3].

The harmful weight loss strategies include severe fasting, fluid restriction, vomiting, laxatives and diuretics consumption, and provoked extreme dehydration by training with plastic/rubber suits or in hot environments, such as saunas [4]. These practices were very well documented in wrestling [5,6] but were extended to other combat disciplines [7,8]. The weight loss cycle presents a very similar pattern when competition is approaching. The cycle consists of a very rapid reduction (3–5 days) reaching in certain cases to 3%–5% of body weight loss, with very rapid subsequent weight gain [9]. All these strategies present many potential risks for the competitor, such as dehydration, fatigue, increased heart rate, headaches, injuries, hyperthermia, loss of consciousness and even death [6]. In addition, the rapid weight changes do not allow an adaptive response of the body, therefore requiring a recovery protocol in order to mitigate the negative effects of rapid weight cycling [10,11].

Altogether, the evidence indicates that a correct nutritional intervention is necessary when planning a long-term weight loss protocol [8,10]. In judo, weight control in national and international tournaments is performed the day before competition and the following day, a maximum increase of only 5% is allowed. If the competitor’s weight is not maintained in this range, he/she is disqualified. Therefore, diet management is a key aspect to reach a certain weight in a specific category as well as to prepare the competition in optimal conditions, including the psychological facet [12,13].

In this particular context, sport scientists and nutritionists need to understand the specific characteristics for weight management in each combat discipline and design healthy strategies to adjust the competitor’s weight while optimizing performance. However, there is a lack of nutritionists in combat sports [13]. This implies that competitors usually manage their weight by themselves or under the advice of coaches and colleagues that are not nutrition professionals. In addition, competitors do not have a background in nutrition, accepting wrong conceptions related to dieting and food control. To this end, the main objective of the present report is to reveal the key aspects related to nutrition that competitors manage and how they plan the weight loss before a national tournament. According to previous publications by other groups and our particular experience, we know that many judo, karate and taekwondo practitioners do not perform appropriate strategies to control their weight before the tournament. Nevertheless, particular aspects regarding management of weight category or particularities of the sport discipline have not been investigated. Therefore, the aim of the study was to obtain information regarding the weight loss strategies that competitors performed before a tournament. Particular attention was given to the volunteer´s knowledge with respect to nutrition, as well as its impact on their health and influence in performance.

## 2. Materials and Methods

### 2.1. Study Design

This is an explorative questionnaire-based study investigating weight loss strategies that competitors performed before a tournament. The comparison between different sport disciplines has been quantitatively analyzed in a previous report [14].

### 2.2. Participants

A sample of 140 male Spanish competitors participated in the study. Volunteers were students randomly recruited from the University National Championship (carried out in 2015) in the following disciplines: judo (*n* = 52), karate-kumite (*n* = 40) and taekwondo (*n* = 48). Mean age of participants was 21.3 ± 2.8 years (range = 18–27 years). Data regarding body composition have been published previously [15]. All weight classes except heavy weights were included in the study.

### 2.3. Questionnaires

Weight control-related aspects were addressed using the RWLQ and EAT-27 questionnaires, which are specific for eating disorders. Validated RWLQ and EAT-27 questionnaires [16,17] were completed before the contest in a national university championship. We believe that the combination of RWLQ and EAT-27 provides a more complete picture than just using one of these questionnaires. In addition, a combination of questionnaires has been successfully applied in other studies [18]. Indeed, a specific questionnaire to cover the eating disorders that occur in combat sports remains to be validated.

The limitation of the RWLQ is that it is more specific for judo. The RWLQ consisted of direct questions (items 1–20). In the case of doubt from participants to answer a specific question, the researcher gave an explanation to the participant adapted to the particular context of the discipline. In any case, doubts were very unusual. The question (item 21) addressing rapid weight loss behaviors presented a scale (always, sometimes, almost never, never used, I do not use anymore) that reflects the grade of harm in the practice [16].

Regarding EAT-27, a previous report indicated that the questionnaire did not address all the critical points for weight control in these sports [14]. Nevertheless, EAT-27 can give some details regarding the psychological relation of the subject with food. On the other hand, EAT-27 (modified by authors from EAT-26 by adding an additional item) included a last question extracted from EAT-40 and related to the use of laxatives and diuretics. EAT-27 also contained scaled questions: never, rarely, sometimes (0 points), often (1 point), usually (2 points) and always (3 points). Items 1 and 25 have their scores inverted [17]. The analysis of internal validity and consistency of the questionnaire (Cronbach’s α) for the 3 factors was: dieting (=0.78), bulimia (=0.82) and oral control (=0.84). Judo: dieting (α = 0.759), bulimia (α = 0.705), oral control (α = 0.736), total (α = 0.788); Karate: dieting (α = 0.749), bulimia (α = 0.754), oral control (α = 0.723), total (α = 0.763); Taekwondo: dieting (α = 0.889), bulimia (α = 0.832), oral control (α = 0.759).

### 2.4. Procedure

All participants voluntarily signed an informed consent. Then, participants filled both questionnaires anonymously, taking approximately 15–20 min. Both questionnaires were completed after weight control and before the first combat in a quiet room under the supervision of a researcher. The study was conducted according to the guidelines of the Declaration of Helsinki and approved by the Ethics Committee of University Miguel Hernandez of Elche (Elche, Spain). 

### 2.5. Data Analysis

SPSS version 27.0 software (IBM Statistics, Armonk, NY, USA) was used for data analysis. Normal distribution of continuous and categorical variables was tested with the Kolmogorov–Smirnov test. Due to the low sample size, descriptive analyses were performed. Associations between variables were performed as contingency tables. The results were verified by means of chi-Square test and the magnitude of the association with the contingency coefficient (CC). Statistical significance was set as bilateral significance (sig) <0.05.

## 3. Results

### 3.1. The Process of Weight Loss in Combat Sports before Competition

Critical points related to unhealthy nutritional practices emerged mainly from three items of the RWLQ; items 17, 20 and 21 [16]. In addition, these items draw a behavioral flow chart when a practitioner of these disciplines decides to lose weight: time in advance (item 17), strategy used (item 21) and how did they know of this strategy (item 20).

#### 3.1.1. Length of the Weight Loss Process

Planning weight loss is an instrumental part of the preparation for the competitive season. For this reason, the first key point was the length of the weight loss process (item 17 in RWLQ). Taking into account all disciplines, half of the participants did not follow a weight loss strategy as they were already in the desired weight. In the rest of competitors, results reported a significant association between the general timeframe to lose weight before a competition and the sport disciplines (sig < 0.01). The majority reported to be trying to lose weight for the previous month before the championship. Specifically, 35% of judo players began their weight loss strategy during the week before the competition, while karate and taekwondo athletes preferred to start a month earlier (Table 1).

#### 3.1.2. Strategies Used for Weight Loss

The most commonly used weight loss strategies are extensively listed in item 21 of RWLQ. Special attention was paid to nutritional-based strategies that were centered in fasting and fluid restrictions (Table 2). Approximately 55% of volunteers followed no strategy, mainly because they were already in their target weight (Table 1) or required minimal adjustments. On the other hand, 33% of participants declared to perform severe fasting centered in food/water restriction, being higher in judo (48%). In addition, fasting was commonly combined with increased exercise in conditions favoring dehydration. A low number of competitors used a healthy diet as part of their weight loss program (only 12%, or 2% in the case of judo). We also found a significant association between nutritional strategies to lose weight and the different combat sports (sig. < 0.05; CC = 0.602).

#### 3.1.3. Source of Information for the Weight Loss Strategy

A key point in the strategy used for weight loss is the role of influencers (item 20 of RWLQ). Participants found influencers in three environments: gyms, family and social contacts (including friends). Fifty-two percent of participants declared no influencer help (Table 3) because they generally reached their category weight before the tournament, confirming results from Table 1 and Table 2. However, around 30% of individuals planned the weight reduction strategy by themselves, mainly by searching for information on the internet or social networks, being more common in judo (40%). The gym represents the second influence environment (almost 15%), being higher in taekwondo (23%). In this particular context, the coach represents the main influencer in all disciplines. Finally, other environments, such as the family, have a very low influence (around 2%). The influence of professionals (nutritionist) is minor, being around 2%. In any case, the associations are not significant, the data of Table 3 being mostly indicators of a tendency.

### 3.2. Strategies to Lose Weight Regarding Competition Category, the Off-Season Body Weight and Proximity of the Contest

Analyzing each individual sport discipline, judo competitors sought the greatest weight losses. In particular, 50% of judo participants of lower weight categories (extra light: less than 60 kg, medium-light: 60–66 kg and light: 66–73 kg) lost an average of 2–5 kg before a contest. This was also observed in 40% of middle weight judo competitors (half middle: 73–81 kg and middle: 81–90 kg). However, the majority of karate and taekwondo practitioners generally needed to lose 0.5–2 kg before contest. Nevertheless, these associations were not significant. To analyze body weight loss strategies depending on the discipline, the off-season body weight was correlated with the weight during competitions. As a result, it was observed that those that competed in a body weight closest to their normal weight had better results in competition. This was the case in two thirds of judo competitors in light (less than 73 kg), 71% in half (73–90 kg) and 100% in heavy (more than 90 kg) weights. Regarding karate, this occurred in 67% of light (less than 67 kg), 65% of middle (67–75 kg) and 89% of heavy (more than 75 kg) weights. In taekwondo, this occurred in 69% of light (less than 68 kg), 70% of middle (68–80 kg) and 70.5% of heavy (more than 80 kg) weights. All associations were significant (sig. < 0.05; CC = 0.364)

In addition, the strategy used to lose weight differed depending to the proximity to the competition. Around 60% of competitors with normal weight close to the weight category in which they generally competed (less than 1 kg difference) did not perform any strategy, besides dehydration before weight control. A proportion of 30% reduced intake or increased exercise the day before weight control (Table 4). If the margin of the usual weight with respect to the category was 1–3 kg, 53% preferred food restriction or to increase exercise, or both. An additional 35% followed a diet under the supervision of a dietitian (Table 4). This last group was mainly recorded in karate and taekwondo competitors. Surprisingly, all judo athletes preferred food restrictions or to increase exercise, or a combination of both strategies. Independently of the discipline, all competitors with usual body weight surpassing 3 kg with respect to their category preferred food restrictions or to increase exercise, or both (Table 4). We found a significant association (sig. < 0.05; CC = 0.243) between the strategy to lose weight and the difference in weight with respect to the competition weight class.

### 3.3. Psychological Aspects Regarding the Eating Behaviour of Competitors

In order to analyze the nutritional information, an EAT-27 questionnaire was completed by the competitors at the same time as the RWLQ. Several key items emerged from the EAT-27 complementing the information obtained from RWLQ. Item 7 (“I particularly avoid foods with a high carbohydrate content (i.e., bread, rice, potatoes, etc)”) was the only one to provide significant results in all combat disciplines. The majority of athletes never (42%) or almost never (24.6%) avoided carbohydrate consumption. However, a significant 33.4% avoided carbohydrate consumption to a certain degree. These results seemed to go in the same way as those obtained in item 16 (“I avoid food with sugar in them”), although they were not significant in this particular item.

**Figure 1:**
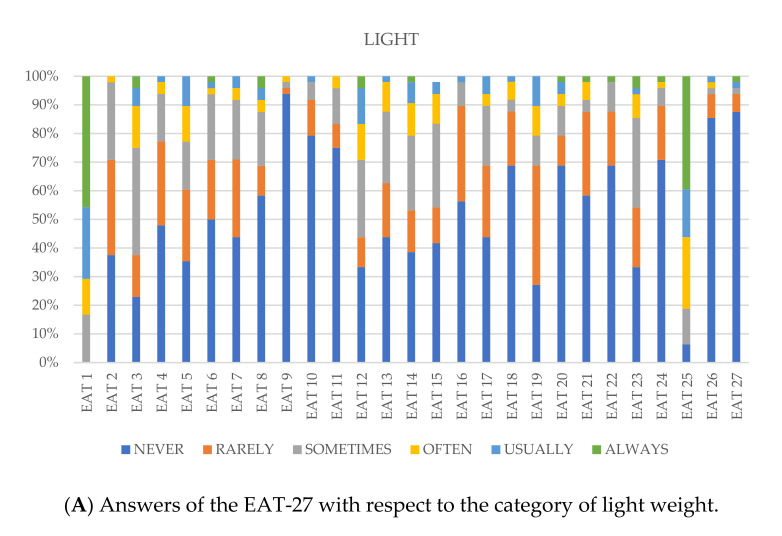

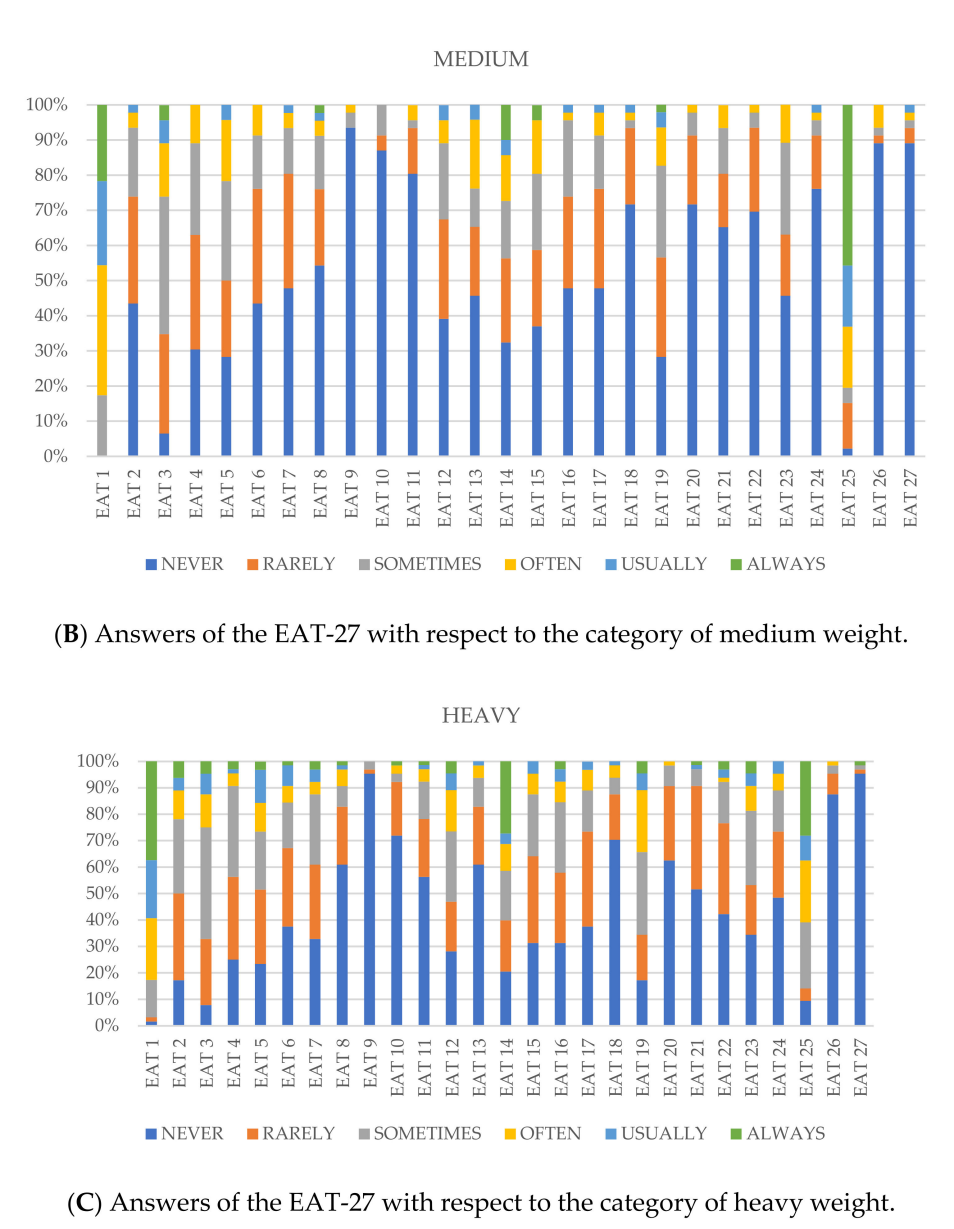
Answers of EAT-27 with respect to different weight categories (light, medium and heavy).

Figure 1A–C show the % response of each option as a function of body weight. For more detail see Table S1. Significant associations are observed in item 2 (sig. = 0.030; CC = 0.461), item 16 (sig. = 0.049; CC = 0.161), item 19 (sig. = 0.024; CC = 0.552) and item 25 (sig. = 0.015; CC = 0.684).

**Figure 2:**
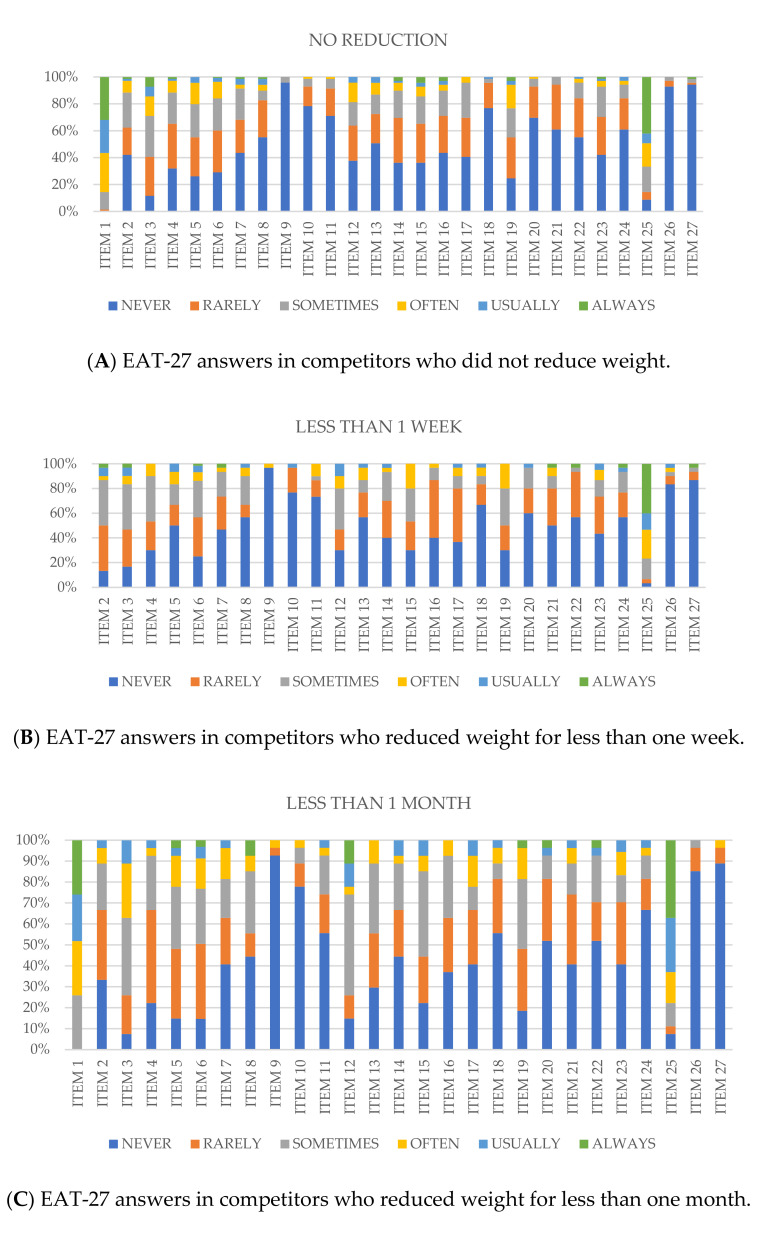

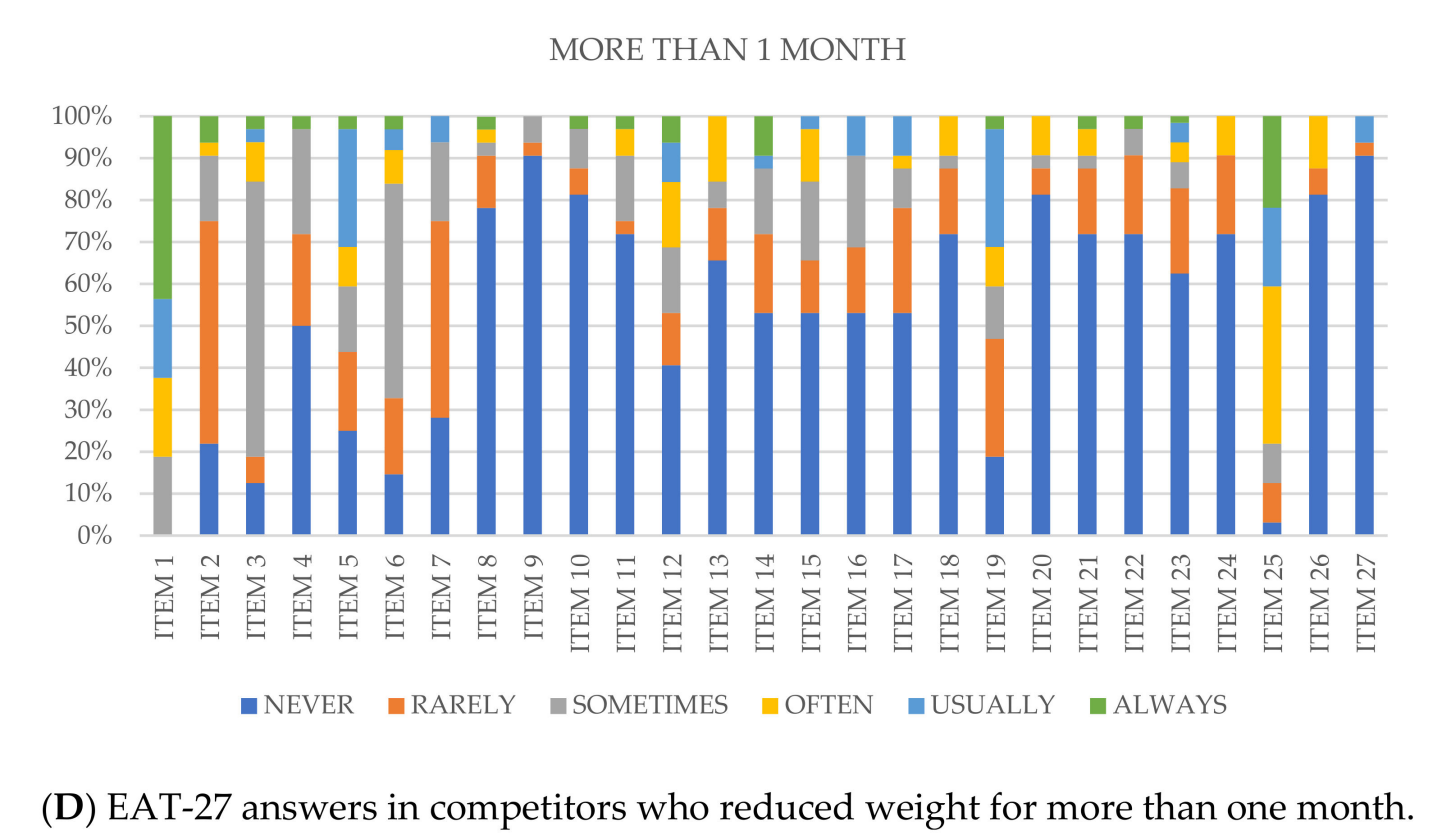
Answers of EAT-27 regarding the time involved for weight reduction (no reduction, less than one week, less than one month and more than one month).

Figure 2A–D show the % responses of EAT 27 depending on the time spent by the players to reduce body weight. For more detail see Table S2-. Significant associations were observed in item 5 (sig. = 0.01; CC = 0.709), item 8 (sig. = 0.023; CC = 0.477), item 12 (sig. = 0.011; CC = 0.569), item 19 (sig. = 0.004; CC = 0.662) and item 20 (sig. = 0.027; CC = 0.536).

As commented previously, an additional item was included in the EAT questionnaire, referring to the use of laxatives and/or diuretics. This question is relevant because the use of these substances is considered as doping in the majority of combat sports. In this context, the majority of participants (91%) declared not taking these substances, and 9% reported occasionally take them.

When the results of the EAT-27 questionnaire were analyzed taking into account the competitor´s weight category, several significant associations were detected. Due to the differences in weight categories among the sports disciplines, it was decided to unify them. Independent of the sports disciplines, all competitors with body weight less than 65 kg were considered light, 65–75 kg were medium, and more than 75 kg were considered heavy. Item 2 (“I avoid eating when I am hungry”), item 16 (“I avoid foods with sugar in them”), item 19 (“I display self-control around food”), item 22 (“I feel uncomfortable after eating sweets”) and item 24 (“I like my stomach to be empty”) received higher scores in the heavy weights (Figure 1A–C). It must be noted that 28% of heavy, 46% of medium and 39% of light weights did not enjoy (item 25) trying new rich foods (Figure 1A–C). Altogether, the answers obtained indicate that individuals in the heavy weight categories were more concerned about their weight control compared to other categories. In this context, results from Figure 1A–C provide a more complete picture than those depicted in Table 1 for judo athletes, where two thirds that lost weight in less than one month but more than one week were included in the medium weight category indicated in Figure 1B (65–75 kg). Meanwhile, 50% and 50% of competitors that tried to lose weight during the last week before the competition were detected in the light and heavy weights, respectively. In karate and taekwondo, these associations were not significant.

In addition, the analysis of EAT-27 provided some significant answers complementing the information obtained in Table 1. Item 8 (“I feel that others would prefer if I ate more”) received a higher score in those that reduced weight during the last week (Figure 2A–D). However, item 11 (“I am preoccupied with a desire to be thinner”) and item 12 (“I think about burning up calories when I exercise”) received higher scores in those individuals that lost weight during the last month before the tournament (Figure 2A–D).

Reviewing each specific sports discipline, 22% of judo players that reduced weight during the last week gave higher scores in item 8 of EAT-27. The same percentage (22%) of judo players that reduced weight during the last week indicated that they never enjoyed trying new foods (item 25). In addition, 67% of karate competitors that reduced weight during the week before the competition gave higher scores in item 8. These competitors did not usually follow a controlled diet plan, and a third generally preferred to induce vomiting (33%) (item 26) and very few took laxatives and diuretics (item 27). No significant associations with EAT-27 items were detected in taekwondo competitors.

As previously presented in Table 2, approximately 12% of participants declared to follow a balanced diet, the majority of them competing in karate and taekwondo. Almost two thirds (62%) answered positively in item 23 (“I engage in eating behavior”). The positive answer to this item is logical when following a diet. Curiously, when a competitor was not competing, and therefore did not need to maintain his/her body weight, 65% of them reported to perform item 4 of EAT-27 (“I have gone on eating binges where I feel that I may not be able to stop”).

## 4. Discussion

Key points related to unhealthy nutritional practices emerged from three items of the RWLQ: time in advance (item 17), strategy used (item 21) and how did they know of this strategy (item 20) (Table 1, Table 2 and Table 3). Regarding item 17 of RWLQ, we can confirm that in general terms, longer lasting strategies are healthier for weight management [19]. In this context, the pre-season period is the best moment to place weight goals and introduce healthy nutritional guidelines [20]. Unfortunately, only 9% of participants that follow a weight loss program do so with sufficient time. This is a point to insist for nutritional education in this segment of practitioners. Fat mobilization, which is the only known strategy for efficient weight loss, takes long periods of time, being particularly difficult in competitors that generally have low fat mass [21]. Therefore, pre-season provides enough time to evaluate the convenience of weight loss and start healthy strategies for weight adjustments, including diet and exercise.

Regarding the strategy used to lose weight (item 21 of RWLQ) (Table 2), the main goal of nutritional education programs is to eliminate unhealthy nutritional practices and explain the risks for health. In addition, these strategies significantly reduced glycogen reservoirs, which are the main source of energy during competition [22,23]. Furthermore, dehydration limits technical execution, accelerates fatigue and puts at risk cardiovascular function, leading to sudden injury and life-threatening conditions [24]. Altogether, the results shown in Table 1, Table 2 and Table 3 reinforce the figure of the sport nutritionist as a main actor to manage healthy weight reduction in combat sports. However, this figure is absent in the majority of cases.

Therefore, in order to obtain the best results in the competition, the data from Table 4 strongly indicate that the optimal weight category should be closest to their normal body weight during the season. A larger weight difference with respect to their weight category generally resulted in a higher tendency to follow unhealthy weight loss strategies. Finally, the answer of item 7 of EAT-27 is worrying, as carbohydrates are the main source of energy in combat sports, and avoiding consumption can dangerously limit the energy of the athletes during the competition [25]. Altogether, the answers from EAT-27 indicate that a significant percentage of competitors generally follow restricted diets with little flavor, making this period of weight reduction (one week) very hard from a psychological point of view. This could have a negative effect on reaching the weight goal by the day of the competition.

The answers obtained from the questionnaires provided a general picture of the behaviors related to dietetic habits that are present in around half of the competitors. The limitation of this study, and others based on questionnaires, is if the answers provided by participants reflect a real situation. A more comprehensive and in-depth study needs to be designed and carried out. Nevertheless, the first noticeable outcome was that unhealthy dietary habits were more prevalent among judo athletes (around 30%). This could be associated with a matter of tradition and experience in this particular discipline. Judo is a very popular combat sport because it was the first Olympic discipline coming from an East Asian martial art and likely is more demanding than the others. An additional limitation is that the Olympic weight classes in the three combat disciplines are not comparable among the three sports disciplines. For instance, heavy weight in Olympic judo corresponds to individuals with more than 100 kg. In Olympic karate, heavy weight starts at 75 kg and in Olympic taekwondo at 80 kg. Therefore, the strategies and mental efforts to reach a certain weight may differ, and this translates to different states of anxiety [26]. 

The most common unhealthy nutritional strategies observed, particularly in judo athletes, included a rapid weight loss (one week) through severe food and water restriction, planned without the control of a professional and following protocols found mainly on the internet, with no scientific basis. The best results were obtained when an individual competed in a weight category closest to their normal off-season weight. The results seemed to indicate that participants identify strategies performed during the last week before the competition unhealthier than those performed with more time in advance, such as one month. In any case, as previously commented, the pre-season is the best moment for weight adjustment. On the contrary, unhealthy practices were more common when the competitor´s off-season body weight was far from the competition weight class. This was mainly observed in light weight classes. In addition, carbohydrates were restricted during weight loss strategies. The answers from the EAT-27 questionnaire reflected a strange relationship with food along with the potential presence of certain bulimic-like behaviors (i.e., item 4 of EAT-27).

Regarding this last point, one of the objectives of the present report was to extract information for educational programs that can be delivered in national federations, training centers and gyms to avoid unhealthy and even life-threatening nutritional practices. These programs are not usually imparted, at least in Spain, for combat sports practitioners. This information would be instrumental for any country where these programs are not regularly available. The present data indicated a significant use of unhealthy nutritional strategies in approximately 30% of competitors (mainly in judo), suggesting that this situation must be addressed with urgency. Furthermore, this is particularly worrying as the individuals analyzed were university students that should have a more solid nutritional knowledge than their non-university counterparts. Finally, the obtained data will help to prepare educational courses in the area of nutrition and its relationship to health status, particularly in teenagers and women that are high risk groups in these particular sport disciplines [27,28].

## 5. Conclusions

Taking together all the data, the following guidelines are proposed for these sports disciplines:

- Select a weight class closest to your usual off-season weight.
- Follow healthy dietetic habits under the supervision of a nutritionist, taking in consideration the necessary time and method to reach the weight goal. The less disturbing period is during pre-season.
- Surpassing the upper limit of the weight class by 200–400 g is acceptable. Under these conditions, running and exercising for one hour before the weight control is sufficient to reach the adequate weight.
- After the weight control, carbohydrates and proteins must be consumed in order to replenish body reserves before the competition. Hydration is also equally important.
- Competitors should have a basic but solid education in nutrition, in order to be independent when taking decisions.

## Figures and Tables

**Table 1:** General timeframe to lose weight before a competition (item 17 of the RWLQ: In how many days do you usually cut weight before competitions?).

	Judo	Karate	Taekwondo	All
Do not perform a weight loss strategy	48.1%	71.8%	34.0%	50.0%
Less than 1 week	34.6%	7.7%	19.1%	21.7%
Less than 1 month	17.3%	15.4%	25.5%	19.6%
More than 1 month	0.0%	5.1%	21.3%	8.7%

The sum of each column gives 100%.

**Table 2:** Nutritional strategies to lose weight.

	Judo	Karate	Taekwondo	All
None	50.0%	61.5%	55.3%	55.1%
Balanced diet	1.9%	18.0%	19.1%	12.3%
Fasting *	48.1%	20.5%	25.5%	32.6%

(*) Fasting includes skipping one or two meals daily or not eating at all, and water restriction. The sum of each column gives 100%.

**Table 3:** Influence of the environment on weight loss.

	Judo	Karate	Taekwondo	All
Category weight	44.2%	61.5%	53.2%	52.2%
Individual itself *	40.4%	28.2%	17.0%	29.0%
Gym **	11.5%	7.7%	23.4%	14.5%
Nutritionist	1.9%	0.0%	4.3%	2.2%
Others ***	1.9%	2.6%	2.1%	2.2%

(*) Internet. (**) Gym includes training partners and coach. (***) This includes family and friends. The sum of each column is 100%.

**Table 4:** Strategies to lose weight vs. difference of weight respect to weight class.

	Weight Near the Upper Limit of the Class (±1 kg)	Weight Surpassing 1–3 kg the Upper Limit of the Weight Class	Weight Passing More Than 3 kg the Upper Limit of the Weight Class	Total
None	58.8%	11.8%	0.0%	55.1%
Diet	10.6%	35.3%	0.0%	12.3%
Reduced intake/ Increased energy expenditure	30.6%	52.9%	100.0%	32.6%

The sum of each column gives 100%.

## Data Availability

The data that support the findings of this study are available from the first author, upon reasonable request.

## Supplementary Materials

The following are available online at www.mdpi.com/article/10.3390/medicina57090897/s1. Table S1: Significant answers of EAT-27 regarding weight categories. Table S2: Significant answers of EAT-27 regarding time involved for weight reduction.
