# Supplementary material for: Weight Loss Strategies in Male Competitors of Combat Sport Disciplines
Source: Medicina (Kaunas). 2021 Aug 28;57(9):897. doi: 10.3390/medicina57090897 (PMC8467103; doi:10.3390/medicina57090897)
Supplement: Supplementary file 1 [file medicina-57-00897-s001.zip › medicina-1317260-supplementary.pdf]

Table S1. Significant answers of EAT-27 regarding weight categories.

		Never			Rarely			Sometimes			Often			Usually			Always		
SIG	EAT items	L	M	H	L	M	H	L	M	H	L	M	H	L	M	H	L	M	H
0.033	2	40.5%	45.9%	16.9%	26.2%	27.0%	28.8%	31.0%	18.9%	30.5%	2.4%	5.4%	11.9%	0.0%	2.7%	5.1%	0.0%	0.0%	6.8%
0.036	16	57.1%	45.9%	28.8%	33.3%	29.7%	28.8%	9.5%	21.6%	25.4%	0.0%	2.7%	8.5%	0.0%	0.0%	5.1%	0.0%	0.0%	3.4%
0.021	19	31.0%	27.0%	18.6%	42.9%	29.7%	16.9%	11.9%	29.7%	32.2%	9.5%	10.8%	25.4%	4.8%	0.0%	1.7%	0.0%	2.7%	5.1%
0.053	22	71.4%	67.6%	39.0%	16.7%	27.0%	35.6%	9.5%	2.7%	16.9%	0.0%	2.7%	1.7%	0.0%	0.0%	3.4%	2.4%	0.0%	3.4%
0.035	24	69.0%	78.4%	45.8%	21.4%	13.5%	25.4%	7.1%	5.4%	16.9%	0.0%	0.0%	6.8%	0.0%	2.7%	5.1%	2.4%	0.0%	0.0%
0.012	25	7.1%	2.7%	10.2%	0.0%	13.5%	5.1%	9.5%	5.4%	27.1%	21.4%	10.8%	20.3%	16.7%	16.2%	10.2%	45.2%	51.4%	27.1%

Weight classes were the following: (L) light (less than 65 kg), (M) medium (65-75 kg) and (H) heavy (more than 75 kg). See text for more details. Abbreviations used: SIG, significance. The sum of each weight (L, M or H) into the same EAT item gives 100%.

Table S2. Significant answers of EAT-27 regarding time involved for weight reduction.

		Never				Rarely				Sometimes				Often				Usually				Always			
SIG	EAT items	NR	-W	-M	+M	NR	-W	-M	+M	NR	-W	-M	+M	NR	-W	-M	+M	NR	-W	-M	+M	NR	-W	-M	+M
0.047	8	55.1%	56.7%	83.3%	0.0%	27.5%	10.0%	8.3%	0.0%	7.2%	23.3%	0.0%	0.0%	4.3%	6.7%	0.0%	0.0%	4.3%	3.3%	0.0%	0.0%	1.4%	0.0%	8.3%	0.0%
0.039	11	71.0%	73.3%	55.6%	66.7%	20.3%	13.3%	18.5%	8.3%	7.2%	3.3%	18.5%	16.7%	1.4%	10.0%	3.7%	0.0%	0.0%	0.0%	3.7%	0.0%	0.0%	0.0%	0.0%	8.3%
0.003	12	37.7%	30.0%	14.8%	16.7%	26.1%	16.7%	11.1%	25.0%	17.4%	33.3%	48.1%	8.3%	14.5%	10.0%	3.7%	16.7%	4.3%	10.0%	11.1%	16.7%	0.0%	0.0%	11.1%	16.7%

Abbreviations used: +M, more than one month; -M, less than one month; NR, no reduction; SIG, significance; -W, less than one week. The sum of each period of time (NR, -W, -M and +M) into the same EAT item gives 100%.
